# Supplementary material for: Exogenous application of hormones and chemicals to mitigate heat stress in strawberry under tropical summer conditions
Source: PLoS One. 2025 Sep 11;20(9):e0331496. doi: 10.1371/journal.pone.0331496 (PMC12425329; doi:10.1371/journal.pone.0331496)
Supplement: Attachment — Submitted filename: Plos one.docx [file pone.0331496.s002.docx]

**Comments for Author**

- This manuscript “mitigation of heat stress using hormones during summer season for survivability of strawberry plants” is very interesting in which Author focused on effect of heat stress on strawberry and its alleviation through Hormones.
- In abstract Author did not mention the levels or concentrations of hormones used to alleviate heat stress.
- Authors required to write novelty statement about their research
- Introduction is insufficient, Author should compare their research with studies of previous researchers
- Hypothesis of study is not clear in introduction section
- In material and methods section too much headings are used by Authors, it is required to write under minimum headings. Particularly second and third heading of the material and methods.
- Why Author used RCBD design? Whereas experiment was conducted under pots.
- Results are written well by authors but still revision is required to improve results. Author should add significance level or p values in results section.
- Graphical presentation also required to improve. Authors need to write in correct manners on axis of graphs.
- Pearson’s correlation diagram is not clear. It is advised to add clear diagram
- In discussion section Author no need to indicate treatments as T1, T2.. etc Moreover, Author needs to write discussion in well mechanized form.
- Conclusion section needs improvement and required to write limitation and future prospects of their study.
- References should be balanced in reference list as well as in manuscript text.
