# Supplementary material for: Exogenous application of hormones and chemicals to mitigate heat stress in strawberry under tropical summer conditions
Source: PLoS One. 2025 Sep 11;20(9):e0331496. doi: 10.1371/journal.pone.0331496 (PMC12425329; doi:10.1371/journal.pone.0331496)
Supplement: Attachment — Submitted filename: Author Responses.docx [file pone.0331496.s003.docx]

**Author Responses to the Reviewer’s Comments**

**Section-A : latest part**

**Editor comments:**

In your Methods section, please provide additional information regarding the permits you obtained for the work. Please ensure you have included the full name of the authority that approved the field site access and, if no permits were required, a brief statement explaining why.

**Author responses:**

No such body is exist at both institutional & country level and no such permission is needed or applicable for conduction of any research work or experiments regarding **ethical considerations of** plant culture and growth

This was added in section 2.1

**Editor comments:**

In the online submission form, you indicated that

"The data underlying the results presented in the study are available from corresponding author on request"

All PLOS journals now require all data underlying the findings described in their manuscript to be freely available to other researchers, either

1. In a public repository,

2. Within the manuscript itself, or

3. Uploaded as supplementary information.

This policy applies to all data except where public deposition would breach compliance with the protocol approved by your research ethics board. If your data cannot be made publicly available for ethical or legal reasons (e.g., public availability would compromise patient privacy), please explain your reasons on resubmission and your exemption request will be escalated for approval.

**Author responses**

All data generated or analysed during this study are included in this manuscript. No more data, other than raw data, which also provided as supplementary file (3 excel file). Pls check declaration section and supplementary file (excel file)

**Editor comments:**

We note that your author list was updated during the revision process. In order to add or remove authors or update the order of the author byline after initial submission, we ask that authors complete an Authorship Change Request form. You may review our full authorship change policy and download the Authorship Change Request form here: https://journals.plos.org/plosone/s/authorship#loc-authorship-changes.

Please fill out all required sections of the form. If you are adding or removing more than 2 authors, you can complete multiple forms and submit as many forms as needed to reflect the updates. Please return the form(s) as an attachment by emailing [plosone@plos.org](mailto:plosone@plos.org) or by uploading it as a submission file labeled with the file type ‘Other’. The form will be reviewed by PLOS staff for approval. Should the changes be approved, we will require written confirmation from all authors confirming that they approve of the changes. Please note that if your manuscript is accepted, we will not be able to complete the publication process without the completed form.

**Author responses**

**This research was conducted under the direct supervision of the co-authors (Prof. Dr. Sharifunnessa Moonmoon & Prof. Dr. Md. Masudur Rahman - where they played role as Supervisor and co-supervisor respectively of the MS research of the first author (Najra-Tan-Nayeem Salwa). Mistakenly we excluded this co-author name**

**Now added even all the related formalities were completed and a humble request would like to made to include this co-author name**

Authorship Change Request form was downloaded, filled up and uploaded in the system

This co-author name were included in everywhere of the related section ( Pls see author name , affiliations , author contribution section)

**Editor comments:**

Please amend your authorship list in your manuscript file to include author Dr. Md. Masudur Rahman.

**Author responses**

This co-author name were included in everywhere of the related section (Pls see author name , affiliations , author contribution section)

**Editor comments:**

Please amend your manuscript to include a reference list. References must be placed at the end of the manuscript and numbered in the order that they appear in the text. For more information on the formatting of references, please visit the author guidelines at: <http://journals.plos.org/plosone/s/submission-guidelines#loc-reference-style>

**Author responses**

Amendment of the reference list was completed. References were placed at the end of the manuscript and numbered in the order that they appear in the text

**Section –B : previous part**

- **Reviewer #1:** Dear authors,

In this work, it has been studied the effects of exogenous application of different compounds on strawberries during field study. A detailed analysis of photosynthetic parameters is provided, and evidence that the treatments contribute for reducing the leaf temperature differential. The research is relevant in the context of alleviating heat stress, which is expected to increase due to climate change.
However, there are some major points that should be addressed. Certain aspects of the methodological setup require further clarification. I also provide a list of minor points for consideration. I see potential in the data acquired by the authors, and I hope my comments help to improve their work.

Kind regards

**Major points**

- **Comments for Author:**

English writing requires deep revision.

- **Author’s Response**

Thank you for your observation. We recognize that certain parts of the manuscript required improvement in English language and expression. To address this, we have thoroughly revised the manuscript to enhance clarity, grammar, and overall readability.

- **Comments for Author:**

Please provide the precise identification (variety) of the tested genotypes, as research reproducibility is fundamental for its acceptance. This information is also very relevant for future studies as, for instance, aiming at understanding why G1 is tolerant, while G2 and G3 are susceptible to heat stress.

- **Author’s Response**

We fully agree that providing the precise identification of the tested genotypes is essential for ensuring reproducibility and facilitating future research. In response, we have specified the exact varieties used in the study: G1= RABI-3, G2= BARI Strawberry-2 and G3= BARI Strawberry-3. This information has been added to the Materials and Methods section (Section 2.2).

- **Comments for Author:**

I think that is necessary a better description of how the G and P lines are related (sections 2.3 and 2.6). I suggest the authors to create a schematic representation.

- **Author’s Response**

We understand the confusion caused by the inconsistent use of labels in Sections 2.3 and 2.6. Specifically, we mistakenly used “P” instead of “G” in some instances when referring to the genotypes. We have now carefully corrected this throughout the manuscript to consistently use “G” (i.e., G1 G2 and G3) for genotypes.

• **Comments for Author:**

In the context of the comment above, it was also not clear for me which variety was used for the treatments. For instance, in Figures 6-18, three varieties are represented on panels b, d and f (P1-3). Do they correspond somehow with the genotype used for the treatments test (panels a, c, and e)?

- **Author’s Response**

We appreciate your attention to the figure layout and labelling. To clarify, the treatments were indeed applied to all three genotypes (G1, G2 and G3). Figures 6-18 were designed to present two separate aspects of the study for each parameter: The effects of genotypes (shown in panels a, c and e) under various treatments and the overall effect of treatments (shown in panels b, d and f) averaged across genotypes.

Thus, both treatment and genotype effects were assessed within the same experimental setup.

Additionally, we acknowledge the labelling error in the genotype panels, where the genotypes were mistakenly labeled as P1, P2 and P3. These have now corrected to G1, G2 and G3 in the figures, figure legends, and relevant sections of the manuscript for clarity and consist

• **Comments for Author:**

Please provide the full details of soil fertilization (concentration and manufacturer, amount and source of the manure, etc), fungicide and pesticide (manufacturer, concentrations).

- **Author’s Response**

We agree that providing detailed information on soil fertilization, as well as the fungicide and pesticide applications, is important for transparency and reproducibility. In response, we have revised the Materials and Methods section (section 2.4) to include the following details:

The soil was mixed thoroughly with fertilizers following the BARC (2012) recommendation. Per pot, 250g vermicompost, 1.67 g TSP, 1.83 g MoP and 0.08 g MgSO_4_ were applied and kept for some days after watering to settle the soil.

• **Comments for Author:**

Please also mention at which time of the day, and at which day of the month the measurements were made. This is important for understanding why some of the observed effects did not appear on the first month of the analysis.

- **Author’s Response**

As mentioned in the revised manuscript, chlorophyll fluorescence data were collected during the first half of the day on three specific dates: 30^th^ March, 30^th^ April and 30^th^ May. These points were strategically chosen to capture plant response under progressively increasing heat stress conditions, thereby allowing us to compare physiological performance across varying temperature intensities over time. We have now clearly stated this information in the Materials and Methods section (section 2.7).

- **Comments for Author:**

The manuscript would benefit from a deeper bibliography input. That goes from the decision for the selection of the compounds for treatments to the analysis of the results. Statements in the Results should be supported by a source reference, even if the information is taken for granted. The impact of heat stress on the photosynthetic apparatus has been greatly studied in other species, often in combination with biotic or chemical stimulants. The deeper bibliographic review would also help to improve the Discussion, which has only 5 cited references.

- **Author’s Response**

Thank you for your insightful suggestion. We fully agree that strengthening the manuscript with a deeper and more comprehensive bibliographic foundation is essential to support our methodological choices and to place our findings within the context of existing research.

In response, we have significantly expanded the literature review in both the Introduction and Discussion sections.

Minor aspects

• **Comments for Author:**

Regarding the experimental setup (point 2.2), the authors mention that the treatments were performed during constant intervals over three months and repeated thrice, between October-November 2023 to April-May 2024. Next (point 2.7), I understand that an initial set was prepared and from this set, the three experiment rounds were started in parallel. However, this point is elusive and I do suggest to clarify it. Was there a time window between the start of each experimental round? If not, I would consider these not as three independent experiments, but rather as one larger experiment with a good number of replicates (Figure 3). While this design can be valuable for statistical analysis, especially in a field experiment, I would still recommend repeating the study to confirm the results.

- **Author’s Response**

To clarify, the three replications mentioned in the manuscript refer to biological replicates arranged simultaneously under the same environmental conditions—i.e., all genotypes and treatments were applied in parallel within a single experimental cycle. There was no time window between the start of each replication. Therefore, we agree that the setup represents one complete experiment with multiple replicates, rather than three temporally independent experimental rounds.

• **Comments for Author:**

Scientific names should be italicizes (e.g. Fragaria, Rosaceae)

- **Author’s Response**

We have reviewed and corrected the formatting of all scientific names, including genus and family names, to ensure they are properly italicized in accordance with scientific writing conventions (Pls see first line of Introduction).

- **Comments for Author:**

Some abbreviations are lacking their full description next to its first mention (e.g. RCBD, PGR; RH-);

- **Author’s Response**

We have carefully revised the manuscript to ensure that all abbreviation, including RCBD, PGR and RH are now presented with their full forms upon first mention (Pls see ABSTRACT). This correction has been applied consistently throughout the text.

• **Comments for Author:**

Please provide the reference for the RCBD model.

- **Author’s Response**

We have now included a reference to support the use of the Completely Randomized Design (CRD) model applied in this experiment. The reference added is:

Gomez, K.A., & Gomez, A.A. (1984). *Statistical Procedures for Agricultural Research* (2nd ed.). John Wiley & Sons.

- **Comments for Author:**

I suggest to move the description of treatments that are now in the section 2.7 to section 2.8, so it would be next to the full description of the treatment application. Also, please provide the manufacturer of each reagent used. I would also suggest to clearly specify the temperature at which the reagents were stored;

- **Author’s Response**

The description of treatments are now in the section 2.5 Specifications of the reagents with stored temperatures were provided (Pls see section 2.5)

- **Comments for Author:**

All Figures would benefit from better legends. Please introduce more details, including full description of what they are representing, description of the error bars, statistical test applied, number of individuals tested per condition (n), etc.

- **Author Response**

All the recorded data were transferred in Microsoft Excel and data curating, sorting, arranging, processing, visualization etc., were performed. Data was then loaded in the R software (R Core Team, 2024) and RStudio ( Version: 2025.05.0+496) to carry out all kinds of statistical analysis. Linear mixed modelling was used to perform ANOVA in the light-dependent parameters. The light-independent parameters were analyzed by performing two-way ANOVA. The mean of the significant parameters were separated using the fisher LSD test (Gomez and Gomez, 1984) at 0.05 level of p-value. The “tidyverse” package was used for the calculation of mean and sem. The data was visualized in graph form using the “ggplot2” package along with others. Pearson’s correlation test were implied in the parameters using the “metan” package along with performing the significant test (Pls see section 2.8).

• **Comments for Author:**

Please also provide the version of each software and R package used.

- **Author’s Response**

In response, we have updated the Materials and Methods section to include the version details of all software and R packages used in the analysis. Specifically, statistical analyses were performed using R software version: 2025.05.0+496 (Pls see section 2.8).

• **Comments for Author:**

I suggest that each treatment be described by both its full name and abbreviation (T1–T5) when presenting the results. This would make the reading easier. Also, please add the description of each treatment o the figures’ legend.

- **Author’s Response**

We have revised the manuscript to present each treatment with both its full name and corresponding abbreviation (T1–T5) for clarity and ease of reading in result section. This includes a clear description of each treatment—5% Molasses (T1), 10 ppm Melatonin (T2), 5% Kaolin (T3), 10 mM CaCl₂ (T4), and 5 ppm Abscisic Acid (T5)—in the text.

• **Comments for Author:**

I suggest to increase the font size of Figure 19 for easier reading. Also, although not really critical, blue and red are usually used for denoting negative and positive values/correlation, so I suggest to swap them to avoid any misinterpretation. I understand that data across all genotypes and treatments were combined and used to plot the Pearson’s correlation. This information should be clear. Furthermore, I do suggest to review the data analysis and interpretation, as for instance the observation that “SPAD increases ambient temperature”, which is unlikely. Still, I wonder whether it might be more informative to represent the correlations of the tested parameters separately for each genotype and treatment. This way one would be able to evaluate their inputs independently.

- **Author’s Response**

God suggestions, Agreed but, sorry, I am not skilled enough thatswhy transformed the respective page into landscape format, so fur readability is increased slightly.

**Reviewer #2: Comments to the author (PONE-D-25-14863)**

The manuscript titled “Mitigation of heat stress using hormones during summer season for survivability of strawberry plants” is an interesting study. The study by Salwa et al is mainly focusing on the use of five exogenous substances (Molasses, Melatonin, Kaolin, CaCl₂, and Abscisic Acid) to mitigate heat stress in three strawberry genotypes under tropical summer conditions which involves physiological and fluorescence measurements across different months. To be honest, the study is interesting and I truly enjoy the reading of manuscript. Overall, the research is valuable and relevant, particularly in the context of climate resilience in horticultural crops. However, some extensive improvements are needed to enhance the scientific clarity, rigor, and writing quality. As per my observation, in the current form, it lacks hypothesis-driven design clarity, and some methodological and interpretational issues need attention. Some suggestions are as follow:
It is extremely embarrassing to review the paper without line numbers to point-out the specific corrections. But I have following suggestions to consider:

- **Comments for Author:**

I would strongly suggest to change the title as: Exogenous Application of Hormones and Chemicals to Mitigate Heat Stress in Strawberry Under Tropical Summer Conditions.

- **Author’s Response**

Good suggestions. Agreed and the title has been changed as per reviewer suggestions

(Pls see latest title)

- **Comments for Author**

Overall, the abstract should be carefully revised as it lacks clarity and scientific soundness. Terms like “some sort of capability” are vague and has incomplete phrasing. Please provide better summary of the most effective treatments and genotype response needed.

- **Author’s Response**

Thank you for your constructive feedback on the abstract. We have carefully revised it to improve clarity and scientific precision. Vague expressions such as “some sort of capability” have been replaced with specific and accurate terminology. Additionally, the abstract now provides a concise summary highlighting the most effective treatments and genotype. These changes aim to better reflect the key findings and improve the overall scientific soundness of the summary.

- **Comments for Author**

In the introduction section, it needs better framing of research gaps. For example, what is unknown about hormone-based mitigation strategies in strawberry under field-like summer stress particularly in Bangladesh. Further, inconsistent tenses and awkward phrasing are present throughout, e.g. “It is establishing climatic change...” is an unclear statement. Further, “…mandatorily has to endure high heat” is overly informal. In Introduction, it doesn’t clearly state the hypothesis and research question as well as key gaps.

- **Author’s Response**

Thank you for your detailed and helpful observations. In response, we have thoroughly revised the Introduction section to improve the clarity, structure, and scientific tone. The framing of the research gap has been strengthened by clearly highlighting the limited understanding of hormone- and compound-based mitigation strategies for managing heat stress in strawberries, particularly summer conditions in tropical regions such as Bangladesh. Specific attention is now given to the lack of research on exogenous treatments like melatonin, kaolin, and abscisic acid in improving heat tolerance in locally grown strawberry genotypes. We have also corrected tense inconsistencies and rephrased unclear or informal statements—for instance, “It is establishing climatic change” and “mandatorily has to endure high heat”—to improve readability and precision. Finally, the revised Introduction now clearly states the research hypothesis and objectives, which focus on evaluating the physiological and biochemical effects of heat stress and assessing the effectiveness of selected treatments across different strawberry genotypes ( Pls see color changed text of INTRODUCTION).

- **Comments for Author:**

Material and methods are the most important section and it should have clear citations/protocol used in the present study. There are some key parts to improve. In this section, genotypes are inconsistently labeled, as sometimes G1/G2/G3, elsewhere P1/P2/P3 there must be uniformity throughout the manuscript. Please use scientific or cultivar names of strawberry genotypes if available and specify the concentration basis for each treatment (e.g., ppm or % w/v, and how these concentrations were finalized/selected. Please provide justification why these 5 treatments were chosen or provide appropriate literature citations/physiological rationales.

- **Author’s response:**

We ensured consistency in genotype labeling throughout the manuscript. All references to genotypes have been uniformly corrected to G1, G2, and G3, and we have also provided their full cultivar names at first mention and we have clarified the concentration basis for each treatment. In addition, we have provided citations and brief physiological rationales for the selection of % or dose etc for each treatment

- **Comments for Author:**

It is unclear that how many number of replicates per treatment/per genotype were used. Was the fluorometer standardized/calibrated before use? Any internal checks? Please elaborate the statistical models in more depth. Was random effect for replication included and interaction terms analyzed?

- **Author’s response:**

Three biological replicates were used per treatment per genotype, resulting in a fully factorial design. Regarding data collection, the fluorometer was standardized prior to each measurement session using the manufacturer's calibration protocol, and internal performance checks were conducted to ensure consistency and accuracy. Although replication was not treated as a random effect due to the CRD design and simultaneous application of treatments, we have noted this as a limitation and suggested it for future model improvements.

- **Comments for Author:**

In the results section, many figures show overlapping trends please consider consolidating. Leaf surface temperature being higher in treatment than control contradicts expectation. Explanation is speculative; check methodology or measurement error. Graph captions needed improvements as some are mislabeled (e.g., gH shown but figure caption mentions φNPQ Fig. 18).

- **Author’s response:**

To address redundancy and improve clarity, we have consolidated figures that showed overlapping trends and provided more concise visual presentations. Regarding the unexpected observation of higher leaf surface temperature in some treatments compared to control, we have rechecked our methodology and instrument calibration. No technical errors were identified; however, we have revised the text to present a more cautious interpretation rather than speculative explanation. This may indicate treatment-induced physiological changes, which we now discussed. Additionally, we have corrected mislabeled figure captions, including the one previously referencing φNPQ instead of gH in Fig. 18. All figure legends have been revised to accurately describe the variables shown.

- **Comments for Author:**

Please provide numerical values with statistical summaries (e.g., mean ± SE) in text for major parameters.

- **Author’s response:**

We have revised the Results section to include statistical summaries, and have now presented the relevant p-values to highlight significant differences among treatments and genotypes.

- **Comments for Author:**

In statistical analysis part, please clarify what variables were considered random vs fixed in LMM. Further, Pearson correlation matrix (Fig. 19) is dense but informative and interpret more in text.

- **Author’s response:**

In our study, treatment and genotype were considered as fixed effects, and replication as a random effect within the linear mixed model (LMM) framework.

Regarding the Pearson correlation matrix (Fig. 19), we agree that while it is dense, it provides valuable insight into the relationships among physiological and biochemical traits. We have now expanded the interpretation in the Results and Discussion sections to highlight key correlations—such as the strong positive association and the negative correlation between the parameters- helping to contextualize these relationships within the framework of heat stress response mechanisms.

- **Comments for Author:**

Overall discussion is descriptive, not mechanistic. Please include some information on the roles of ABA in stomatal regulation under heat, Melatonin as antioxidant/protective compound, Kaolin and molasses as physical shields against radiation, and also genotype differences are needed to be discussed. Please provide recent literature and add more context from global strawberry stress physiology.

- **Author’s response:**

Done, Revised and new references were included

- **Comments for Author:**

Conclusion Needs to state clear practical recommendations, e.g., which combination(s) are best for growers. It must be concise to the point. Also, clarify that this is a first step, and in future long-term, field-based, and reproductive/yield trait studies are needed. add these as future directions.

- **Author’s response:**

In response, we have revised the conclusion to emphasize specific combinations were most beneficial under heat stress and are practical for growers to consider. We have also clarified that this study represents a preliminary step, and highlighted the need for future long-term, field-based studies incorporating reproductive and yield-related traits to validate these physiological findings under real-world conditions.

**Reviewer #3:**

This manuscript “mitigation of heat stress using hormones during summer season for survivability of strawberry plants” is very interesting in which Author focused on effect of heat stress on strawberry and its alleviation through Hormones.

- **Comments for Author:**

In abstract Author did not mention the levels or concentrations of hormones used to alleviate heat stress.

- **Author’s response:**

We agree that mentioning the concentrations of the hormones used is essential for clarity and reproducibility. In response, we have revised the abstract to include the specific concentration of all treatments applied to alleviate heat stress: 5% Molasses (T1), 10 ppm Melatonin (T2), 5% Kaolin (T3), 10 mM CaCl­_2_ (T4) and 5 ppm Abscisic acid (T5).

- **Comments for Author:**

Authors required to write novelty statement about their research

- **Author’s response:**

We have now included a clear novelty statement in the revised manuscript to highlight the unique contributions of our research. Specifically, this study is the first to evaluate and compare the effects of five different exogenous substances—molasses, melatonin, kaolin, calcium chloride, and abscisic acid—on mitigating heat stress in strawberry genotypes under tropical, field-like summer conditions in Bangladesh. The integration of physiological and biochemical responses with statistical modeling provides new insights into treatment-specific and genotype-specific heat tolerance mechanisms.

- **Comments for Author:**

Introduction is insufficient, Author should compare their research with studies of previous researchers

- **Author’s response:**

In response, we have thoroughly revised the introduction section to better contextualize our study within the existing literature. We have included detailed comparisons of key findings related to ABA’s role in stomatal regulation under heat stress, melatonin’s antioxidant function, and the use of kaolin and molasses as physical protectants. Furthermore, we have emphasized the research gaps regarding genotype-specific responses and the integration of biochemical and physical treatments under heat stress conditions. This revised introduction now clearly demonstrates the novelty and relevance of our work in advancing the understanding of heat tolerance mechanisms in strawberry cultivation.

- **Comments for Author:**

Hypothesis of study is not clear in introduction section

- **Author’s response:**

In response, we have revised the introduction section to explicitly state the hypothesis guiding this research.

- **Comments for Author:**

In material and methods section too much headings are used by Authors; it is required to write under minimum headings. Particularly second and third heading of the material and methods.

- **Author’s response:**

This revision reduces unnecessary fragmentation and presents the methodology more clearly under fewer, well-organized headings.

- **Comments for Author:**

Why Author used RCBD design? Whereas experiment was conducted under pots.

- **Author’s response:**

Yes, the experiment was conducted using potted plants. Therefore, the appropriate experimental design is the Completely Randomized Design (CRD), not the Randomized Complete Block Design (RCBD) as was mistakenly mentioned in the original manuscript. We have now carefully corrected this error.

- **Comments for Author:**

Results are written well by authors but still revision is required to improve results. Author should add significance level or p values in results section.

- **Author’s response:**

In response, we have revised the results to incorporate appropriate significance levels and p-values for all relevant comparisons, clearly indicating which differences are statistically significant.

- **Comments for Author:**

Graphical presentation also required to improve. Authors need to write in correct manners on axis of graphs.

- **Author’s response:**

We acknowledge the importance of clear and accurate axis labelling for proper data interpretation and have carefully revised all figures to address these concerns. Specifically, we have ensured all axes now clearly.

- **Comments for Author:**

Pearson’s correlation diagram is not clear. It is advised to add clear diagram

- **Author’s response:**

we have recreated the diagram with improved resolution, clearer labels to enhance interpretability.

- **Comments for Author:**

In discussion section Author no need to indicate treatments as T1, T2.. etc Moreover, Author needs to write discussion in well mechanized form.

- **Author’s response:**

We have carefully revised manuscript to address and now used full descriptive names with abbreviation throughout to enhance clarity. We have substantially strengthened the mechanistic discussion by explicitly linking our findings to underlying biological processes, supported by current literature. The discussion section has been reorganized to flow logically from key observations to their mechanistic basis and broader implications.

- **Comments for Author:**

Conclusion section needs improvement and required to write limitation and future prospects of their study.

- **Author’s response:**

we have improved the conclusion section by summarizing the key findings more clearly and added the limitations of our study, such as the controlled environment conditions and limited number of genotypes tested. We also included future prospects, highlighting the need for field trials.

- **Comments for Author:**

References should be balanced in reference list as well as in manuscript text.

- **Author’s response:**

We have carefully reviewed the manuscript and the reference list to ensure a more balanced and comprehensive citation of relevant recent and foundational studies throughout the text.

**Reviewer #4:**

In the present manuscript, the authors evaluated the effect of different PGRs sprays on three different strawberry genotypes to observe the tolerance response of plants from the onset of tropical summer to peak summer. The experimental approach is well justified and well described; however, the results are not adequately presented. In the result sections 3.2, 3.3 (number repeated in this section) and 3.4, there is no indication of which phenotype the data for the different treatments are (Figures, a, c, & e) or from which treatment the data in the phenotype graphs are (Figures, b, d & f). All of the above does not allow for understanding the interpretation of the results or their discussion.
Other minor concerns are:

- **Comments for Author**

All abbreviations must be defined in the abstract and when they are first used in the introduction or materials and methods sections (RCBD, RH, SPAD, PGRs…)

- **Author’s Response**

We have carefully revised the manuscript to ensure that all abbreviation, including RCBD, RH, SPAD and PGRs are now presented with their full forms upon first mention. This correction has been applied consistently throughout the text.

- **Comments for Author**
  P1, P2 & P3 must be G1, G2 & G3 in section 2.6 of MM
- **Author’s Response**

we mistakenly used “P” instead of “G” in some instances when referring to the genotypes. We have now carefully corrected this throughout the manuscript to consistently use “G” (i.e., G1 G2 and G3) for genotypes.

- **Comments for Author**

In the result sections 3.2, 3.3 (number repeated in this section) and 3.4, there is no indication of which phenotype the data for the different treatments are (Figures, a, c, & e) or from which treatment the data in the phenotype graphs are (Figures, b, d & f).

- **Author’s Response**

We acknowledge the oversight in labeling the phenotypes and treatments in Figures (a, c, e) and (b, d, f), respectively, as well as the unintended repetition in section numbering. To address these concerns, we have now clearly labeled all phenotypes (including units) in the treatment and genotype comparison figures separately, and corrected the section numbering to avoid duplication.

- **Comments for Author** In the MM section, three phenotypes are indicated: G1 (heat tolerant), G2, and G3 (heat susceptible). However, all the corresponding graphics are labeled with the P1, P2, and P3 varieties. Please clarify. These labels are repeated in all the results sections and in the discussion or conclusion sections.
- **Author’s Response**

We mistakenly used “P” instead of “G” in some instances when referring to the genotypes. We have now carefully corrected this throughout the manuscript to consistently use “G” (i.e., G1 G2 and G3) for genotypes.

- **Comments for Author**

Finally, I recommend that a native English speaker revise the entire manuscript

- **Author’s Response**

To improve the clarity and readability of the manuscript, we have revised the manuscript using GRAMMARY software and then arranged for a native English speaker to thoroughly review and edit the entire text. This revision ensures that the language is clear, precise, and free of grammatical errors. We believe this enhances the overall quality of the manuscript.
