# Supplementary material for: Exogenous application of hormones and chemicals to mitigate heat stress in strawberry under tropical summer conditions
Source: PLoS One. 2025 Sep 11;20(9):e0331496. doi: 10.1371/journal.pone.0331496 (PMC12425329; doi:10.1371/journal.pone.0331496)
Supplement: Attachment — Submitted filename: accept.docx [file pone.0331496.s004.docx]

Author have made changes accordingly in good manner. Thus, decision is accept
