# Supplementary material for: Exogenous application of hormones and chemicals to mitigate heat stress in strawberry under tropical summer conditions
Source: PLoS One. 2025 Sep 11;20(9):e0331496. doi: 10.1371/journal.pone.0331496 (PMC12425329; doi:10.1371/journal.pone.0331496)
Supplement: Attachment — Submitted filename: Response to reviewer.docx [file pone.0331496.s005.docx]

**Author Responses to the Additional Editors Comments**

**[Dear Sir,**

**Sorry, I tried several time but failed to build PDF appropriately. I observed that, during PDF formation / build figure -18 is converting figure 17, seems to be repetition, though content is is not changing**

**Therefore total figure number is changing in to 19, instead of 20]**

**Would like to request to consider the fact or technical error ]**

1. **Additional editor comments:**

Kindly see the comments from a reviewer especially Reviewer 4. There is a serious issue on labeling the figures and given proper legends or methodological explanation. Kindly correct the same.

**Author Responses:**

We sincerely thank Reviewer 4 for the valuable feedback. We acknowledge the concern regarding figure labeling and the lack of proper legends or methodological explanations. In response, we have thoroughly revised the manuscript to ensure that:

•All figures are correctly labeled with consistent numbering and appropriate captions.

•Figure legends now provide clear and sufficient detail to be understood independently.

**Author Responses to the Reviewer’s Comments**

**Reviewer #4:**

**1.Reviewer’s Comments**

 The authors have greatly improved the manuscript's language but some revision is still needed.

**Author’s Response**

We have carefully reviewed the entire text once again and made further revisions to enhance clarity, grammar, and overall readability. All changes have been highlighted in the revised version of the manuscript for ease of reference.

**2. Reviewer’s Comments**

However, my concerns remain regarding the presentation of results as explained in the first review.

**Author’s Response**

**The previous comments of reviewer-4** r**egarding results** in the first review **was like below**

**“**The experimental approach is well justified and well described; however, the results are not adequately presented. In the result sections 3.2, 3.3 (number repeated in this section) and 3.4, there is no indication of which phenotype the data for the different treatments are (Figures, a, c, & e) or from which treatment the data in the phenotype graphs are (Figures, b, d & f). All of the above does not allow for understanding the interpretation of the results or their discussion”.

Thanks reviewer for above valuable suggestions

In results (section 3.2 and 3.3) repetition was avoided carefully. In 3.2 the ambient temperature of the leaf surface (Fig. 6), leaf internal temperature (Fig. 7) and the differential temperature (Fig. 8) were described in control and treatment groups of the study

The findings were described properly with clarity. Pls see the color changed text (red color) in section 3.2 (line number 335-342; 344-348; 355-357)

In section 3.3, two important leaf attributes like leaf thickness (Fig. 9) and leaf greenness (SPAD) (Fig. 10) were stated clearly. Please see color changed text (red color) where important findings were highlighted (line number 373 – 377; 386 – 391)

In section 3.4, figure denotes the treatment variation of maximum quantum efficiency where, in March ABA showed the best performance but with the time of increasing temperature (April and May) Kaolin (T3) treatment showed the better performance. In case of figure b. d, f, G1 showed the maximum quantum efficiency compared to the other genotypes (Pls see the line 395- 399 ; 399 -401)

**3. Reviewer’s Comments**

The graphs in Figures 6 through 18 (the number in Figure 17 is repeated) represent the different parameters recorded (Ambient temperature on the leaf surface, Leaf internal temperature, Leaf thickness, SPAD, etc., for each treatment (T0-T5)). The primary concern is that authors do not specify whether the data in the graphs in sections a, c, and e of each figure correspond to all plants or a specific variety.

**Author’s Response**

Thanks. Repetition of figure 17 is avoided

Yes, now specified

Figure 17: Steady-State Proton Flux (vH) of PSII of Strawberry plants under different treatments

and genotypes. Treatment and genotypes having different letter varies significantly at

p ≤ 0.05

and

Figure 18: Proton conductivity (gH) of PSII of Strawberry plants under different treatment and

genotypes. Treatment and genotypes having different letter varies significantly at

p ≤ 0.05

The data in the graphs in sections (3.2 & 3.3) a, c, and e of each figure (8, 9) correspond to all plants. Pls see color changed text (line number 335 and 367)

**4. Reviewer’s Comments**

Furthermore, the graphs in sections b, d, and f of each figure, which refer to the three varieties used, do not specify which treatment (T0-T5) the data are from. The authors should present the data collected for each Strawberry variety and each treatment used to allow for better interpretation, comparison, and discussion of the results.

**Author’s Response**

To clarify, all treatments were indeed applied across all three genotypes (G1, G2, and G3). Figures 6–18 were intentionally designed to illustrate two complementary perspectives for each parameter:

- Panels a, c, and e show the effects of different genotypes under various treatments.
- Panels b, d, and f show the overall effects of treatments averaged across genotypes.

In other words, both genotype and treatment effects were analyzed within the same experimental setup. To generate these visualizations, we created two separate pivot tables from the raw data for each parameter—one summarizing data by genotype and the other summarizing data by treatment.
